# Detection of ALDH1 activity in rabbit hepatic VX2 tumors and isolation of ALDH1 positive cancer stem cells

**DOI:** 10.1186/s12967-016-0785-0

**Published:** 2016-02-12

**Authors:** Prashasnika Gehlot, Vivek Shukla, Sanjay Gupta, Paul E. Makidon

**Affiliations:** Department of Surgical Oncology, The University of Texas MD Anderson Cancer Center, Houston, TX 77030 USA; TGIB Branch, NCI, Betehsda, MD USA; Department of Diagnostic Radiology-Imaging, The University of Texas MD Anderson Cancer Center, Houston, TX 77030 USA; The Unit for Laboratory Animal Medicine, University of Michigan Medical School, Ann Arbor, MI 48109 USA

**Keywords:** Hepatocellular carcinoma, Aldehyde dehydrogenase, Cancer stem cells, Rabbit VX2

## Abstract

**Background:**

Aldehyde dehydrogenase 1 (ALDH1) activity has been implicated in the therapeutic drug resistance of many malignancies and has been widely used as a marker to identify stem-like cells, including in primary liver cancer. Cancer stem cells (CSCs) are thought to play a crucial role in cancer progression and metastasis. In order to clarify the validity of the rabbit VX2 liver cancer model, we questioned if it expresses ALDH1 as a potential marker of CSCs. Hepatocellular carcinoma is a common malignancy worldwide and has poor prognosis. Most of the animal models used to study hepatocellular carcinoma are rodent models which lack clinical relevance. The rabbit VX2 model is a large animal model useful for preclinical and for developing drugs targeting cancer stem cells.

**Materials and methods:**

We used flow cytometry to identify rabbit VX2 liver tumor cells that express ALDH1A1 activity at a high level and confirmed the results with RT-PCR, immunohistochemical and western blot analyses. Further, mRNA and protein expression analysis of tumor samples also express the markers for stemness like klf4, oct3/4, CD44 and nanog as well as the differentiation marker α-fetoprotein.

**Results:**

We used Aldefluor flow cytometry-based assay to identify cells with high ALDH1 activity in the rabbit VX2 liver cancer model. We used the brightest 4.39 % of the total cancer cell population in our study. We performed semi-quantitative as well as real time PCR to characterize the stemness derived from VX2 tumors and tissues from normal rabbit liver. We demonstrated that VX2 tumors have higher expression of cancer stem cell markers such as AlDH1A1 and CD44 in comparison to normal rabbit liver cells. Additionally, real time PCR analysis of the same samples using syber-green demonstrated the significant change (p > 0.05) in the expression of genes. We validated the gene expression of the stemness markers by performing western blot and immunofluorescence. We showed that cancer stem cell markers (AlDH1A1, CD44) and the differentiation marker α-fetoprotein were upregulated in VX2 tumor cells. The same extent of upregulation was observed in stemness markers (klf4, oct3/4 and nanog) in VX2 tumors in comparison to normal rabbit liver.

**Conclusion:**

The overall results of this study indicate that ALDH1 is a valid CSC marker for VX2 cancer. This finding suggests that the rabbit VX2 liver cancer model is useful in studying drug resistance in hepatocellular carcinoma and may be useful for basic and preclinical studies of other types of human cancer.

**Electronic supplementary material:**

The online version of this article (doi:10.1186/s12967-016-0785-0) contains supplementary material, which is available to authorized users.

## Background

Hepatocellular carcinoma (HCC) ranks among the most common and deadly malignancies worldwide. The annual incidence of this disease is an estimated 6,00,000 to 1 million the number of new cases per year is expected to increase over the next few decades because of the high incidence of chronic hepatitis C (HCV) and hepatitis B (HBV) viral infections. There is a large pool of people who are infected with HCV, HBV, or both in whom the cancer is in the latency period. In addition, emigration from areas where hepatocellular carcinoma is endemic, such as Southeast Asia and parts of Africa, to the west where perinatal HBV infection and exposure to environmental carcinogens such as aflatoxin (in particular Aflatoxin B1) are common, is likely to continue. These factors are therefore likely to increase the incidence of HCC in the US [1]. Other factors influencing the incidence of HCC in western societies include nonalcoholic fatty liver disease (NAFLD) and nonalcoholic steatohepatitis (NASH) [2]. Surgical removal of the liver cancer or liver transplantation is the most effective therapies for HCC. However, most patients present with unresectable disease due to poor liver function and not meeting transplantation criteria. Additionally, there is a paucity of donor organs for transplantation. Novel approaches to treating nonresectable HCC are available but are rarely curative, and the 3-year patient survival rate remains relatively low (30–40 %) [3, 4].

Cancer stem cells (CSCs) have been identified as important mediators in leukemia [5], pancreatic [6], lung [7], breast [8] and hepatocellular carcinoma [9] among other malignancies. These cells typically constitute 1–5 % of the total tumor cell population, although some malignancies, such as breast cancer and glioblastoma, have larger CSC populations (11–35 % and 5–30 %, respectively) [10]. CSCs are putatively considered the primary cells responsible for tumor initiation, growth, and metastasis [11–13].

CSCs are believed to have properties similar to their nonmalignant stem cell counterparts such as self-renewal, anchorage-independent growth, and the potential for differentiation [14, 15] into heterogeneous tumors [16, 17]. CSCs are thought to be chemoresistant, which may explain why they are enriched in residual tumors and can, subsequently, mediate tumor recurrence following cytotoxic chemotherapy [18, 19]. For these reasons CSCs are considered attractive chemotherapeutic targets.

The lack of consensus on definitive stem cell markers has compromised the ability to functionally characterize them. Despite these setbacks ALDH1 is emerging as a universal functional marker of choice for cancer stem and progenitor cells [20]. The enzymatic activity of aldehyde dehydrogenase 1 (ALDH1) has been identified as one of the mechanisms for the resistance of progenitor cells to chemotherapeutic agents [21]. The ALDH1 family of enzymes are cytosolic isoenzymes that are responsible for oxidizing intracellular aldehydes and contributing to the oxidation of retinol to retinoic acid in early stem cell differentiation [22]. This enzymatic activity can be used to select for highly enriched populations of progenitor cells in bone marrow [23] and umbilical cord sources [24]. Recent studies have shown that ALDH1 is a CSC marker and that its presence strongly correlates with tumor malignancy as well as the self-renewal capacity of stem cells in tumors of the lung [25], breast [26], colon [27] and hepatocellular cancer (HCC) [28]. Stem cell activity of ALDH1 positive cells has been demonstrated in several different models of cancer such as mammary carcinoma [29], acute myeloid leukemia [30], pancreatic cancer [31], lung cancer [32], ovarian cancer [33] and hepatocellular carcinoma [34]. Our aim in the present study was to determine whether the rabbit orthotopic liver VX2 tumor model could be used as a model for human HCC. The presence of CSCs in rabbit VX2 tumors is hitherto unknown. The orthotopic VX2 tumor is a hypervascular tumor [35]. Using several techniques, we identified ALDH1 activity and the presence of CSC-like cells in VX2 tumors, which suggested that this rabbit model may serve as a preclinical model for investigation of therapeutic stem cell targets in human HCC, closing the gap between the bench and the clinic.

## Methods

### Cell culture

VX2 liver tumor cells from harvested tumors were cultured in DMEM-F12 medium (Gibco Invitrogen, Carlsbad, CA, USA) with 1 % penicillin and 1 % streptomycin (Gibco invitrogen) in an incubator with normoxic atmosphere and 5 % CO_2_ at 37 °C. Single-cell suspensions of tumor cells were prepared by mincing primary rabbit VX2 tumors with a razor blade, washing them, and digesting them in 0.1 % Type I and IV collagenase (Sigma-Aldrich, St. Louis, MO, USA) in DMEM-F12 at room temperature for 15–30 min. A pipette was used to break up clumps in the digested material, and the suspension was visually inspected under a light microscope to confirm that most cells had been released. Remaining clumps were removed by filtration through a 40-μM nylon mesh screen.

### Animals and tumor induction

The studies performed on live animals were approved by the Institutional Animal Care and Use Committee at the University of Texas MD Anderson Cancer Center. Ten adult New Zealand white rabbits (mean weight, 4.04 ± 0.31 kg) were purchased from Charles River Laboratories (Wilmington, MA). The animals were maintained in facilities accredited by the Association for Assessment and Accreditation of Laboratory Animal Care International and in accordance with regulations and standards of the U.S. Department of Agriculture, the U.S. Department of Health and Human Services, and the National Institutes of Health.

### Anesthesia and antibiotics

Anesthetic induction was achieved using isoflurane (0.5–5 %)/oxygen (1.5 L/min) administered via mask. Maintenance anesthesia was accomplished using isoflurane (0.5–5 %)/oxygen (1.5 L/min) administered via mask or, in some cases, an endotracheal tube. Thermal-regulation was maintained for anesthetized animals using a Bair Hugger warming blanket. One peri-operative dose of antibiotics (enrofloxacin; 5 mg/kg) was given I.M.

### Analgesics

Each rabbit receives a single dose of SR buprenorphine pre-operatively (0.1–0.3 mg/kg, followed by PRN/every 72 h as determined by clinical veterinarian). Animals were assessed daily and analgesics were continued for as long as necessary to control pain.

For tumor implantation, each animal was pre-medicated with an intramuscular injection of buprenorphine (0.15 mg/kg body weight; Bedford Laboratories, Bedford, OH, USA), and a surgical plane of anesthesia was maintained using isoflurane. After routine surgical preparation, the left lateral lobe of the liver was isolated following a mid-line abdominal incision. A 2–4 mm section of the donor VX2 tumor was surgically implanted in a focal spot in the left lateral liver lobe [4, 5].

### Aldefluor assay

To isolate cells with high ALDH1 enzymatic activity, we used the Aldefluor kit (Cat. No. 1700, Stem Cell Technologies, Vancouver, BC), which is designed for optimal identification and isolation of stem cells through specific interactions with human ALDH1 (90 % identity with rabbit ALDH1 with the nucleotide-nucleotide blast sequence alignment) (Ginestier, 2007). Cells were stained with Aldefluor reagent and quantified by flow cytometry. The experiments were carried out according to the manufacturer’s instructions. Each experiment was repeated three times. Fresh cells were stained with 1 g/mL propidium iodide (PI; Sigma) for 5 min to determine viability. The gate for ALDH-positive cells were based on diethylaminobenzaldehyde (DEAB)-mediated inhibition of ALDH1, and the gate for viable cells was based on PI exclusion.

### Real-time PCR analysis (mRNA expression)

Total RNA was extracted using Qiagen’s RNeasy mini kit (Cat No. 74104). For preparation of cDNA, we followed the protocol recommended by BioRad (iScript^TM^ cDNA Synthesis Kit, Cat No. 170-8890) by using 500 ng RNA from each sample. Real time PCR analysis was performed using SsoFast^TM^ EvaGreen Supermix (BioRad Cat no. 172-5200). The following primers were analyzed for mRNA expression as shown in (Table 1).

**Table 1:** Primer pairs of RT-PCR experiment

Gene name	NCBI reference sequence	Primer sequence (forward 5′–3′)	Primer sequence (reverse 5′–3′)	Product size (bp)
nanog	XM_002712762.1	GCCAGTCGTGGAGTAACCAT	TGTGCTGTGTTCTGGCTTTC	196
oct3/4 (Pou5f1)	NM_001099957.1	GAGATTTGCAAAGCGGAGAC	CGGTTACAGAACCACACA CG	188
CD44	FJ360436.1	CCACGTGGAGAAAAATGGTC-	CACGTGCCCTTCTATGAACC	157
klf4	XM_008255400.1	TTCAACGACCTTCCTGAACC	TCGGGGTAACCTGAAAACTG	184
Gapdh	NM_001082253.1	AGGTCATCCACGACCACTTC	GTGAGTTTCCCGTTCAGC C	202
Nestin	XM_008264301.1	GTGAGTTTCCCGTTCAGC C	CTCCACATCAGAAGCAGCAA	176
Aldh1a1	AY038801.1	CTACCATCAGGGCCAGTGTT	CCCCTTCTTTCTTCCCACTC	209

### Immunohistochemical analysis

A fluorescence-activated cell sorting (FACS)-selected population of ALDH1-positive VX2 cells were fixed with 4 % paraformaldehyde for 15 min and blocked in 10 % normal serum for 30 min. The cells were incubated with primary anti- Aldh1a1 (Catalog No. # ab23375); CD44 (Catalog No. #ab6124, abcam) and α-fetoprotein (Catalog No.3 2019-08, DAKO) at a working dilution of 1:200, overnight at 4 °C. The cells were then incubated for 1 h with Alexa Fluor-conjugated (anti rabbit) secondary antibody in the dark at 37 °C. Cells were counterstained with 4′,6-diamidino-2-phenylindole (DAPI) in mounting medium (Vector Laboratories, Inc., Burlingame, CA, USA) to demonstrate the presence of nuclei. DAPI-stained negative controls were stained without the primary antibody. Stained cells were examined under a Nikon fluorescent microscope and a Zeiss LCM confocal microscope.

A sample of each tumor (induced as stated earlier) was fixed with 10 % neutral buffered formalin and embedded in paraffin for histopathologic examination. The cut sections (5–7 μm) were stained with hematoxylin and eosin using standard protocols.

### Western blot analysis

Whole-cell proteins were extracted from unsorted cells at 70–80 %. Cells were subjected to lysis in a buffer containing 20 mmol/L Tris–HCl (pH 8.0), 137 mmol/L NaCl, 1 % Triton X-100, 1 mmol/L Na_3_VO_4_, and 2 mmol/L ethylenediaminetetraacetic acid (EDTA) plus one complete Mini Protease Inhibitor Cocktail Tablet (Roche Diagnostics, Indianapolis, IN, USA) per 10 mL of lysis buffer. After we determined the protein concentration of each lysate with the Bradford assay [Bradford M1976], lysate samples containing 40 μg of whole-cell protein extract, 20 μg of ALDH-positive cell proteins, or 20 μg of ALDH-negative cell proteins were subjected to electrophoresis on sodium dodecyl sulfate–polyacrylamide gels (SDS-PAGE) [Laemmli UK., 1970] at a 10 % concentration (based on target protein size) and transferred to polyvinylidene difluoride membranes [6]. Membranes were probed overnight at 4 °C with the primary rabbit Aldh1a1 (Catalog No. # ab23375); klf4 (Catalog No. #4038S, Cell Signaling): oct3/4 (Catalog No. #2750S, Cell Signaling); Nanog (Catalog No. #ab21624, abcam); α-fetoprotein (Catalog No. 3 2019-08, DAKO) and β-actin (Catalog No. ab8227, abcam) at a working dilution of 1:500, washed three times for 10 min each in Tris-buffered saline solution with 0.1 % Tween-20, and probed with secondary antibody (HRP-labeled, goat anti-rabbit IgG [H+L]; Abcam, Cambridge, MA, USA) for 1 h at room temperature at a working dilution of 1:2000. After incubation and three washes, immunostained proteins were detected using a chemiluminescence kit (Thermo Scientific, Waltham, MA, USA). To confirm equal loading, membranes were re-probed with beta-actin antibody. All experiments were repeated at least three times.

## Results

**Fig. 1:**
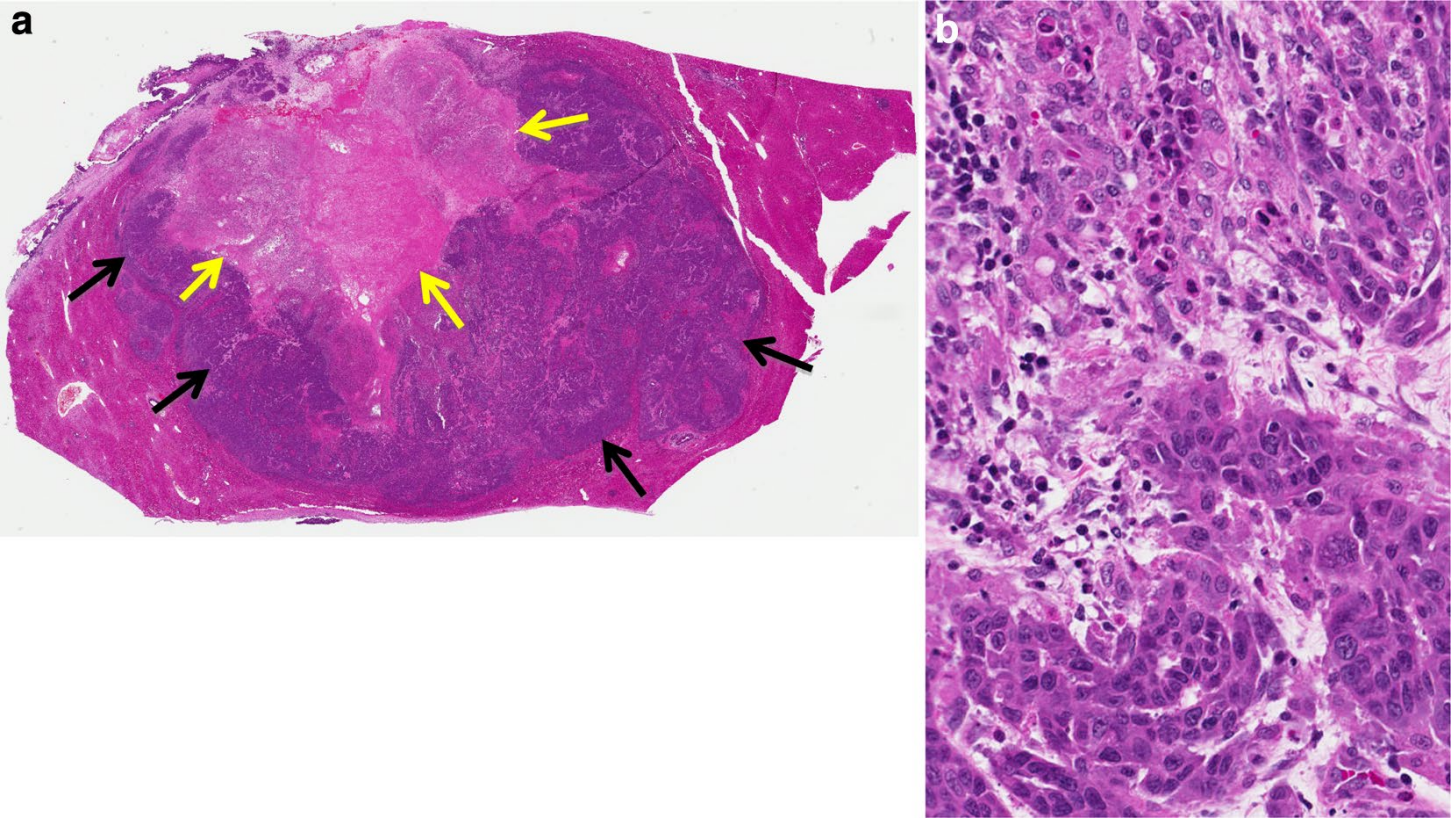
Histological analysis of rabbit hepatic VX2 tumors. **a** Overview of the left lateral liver lobe showing the tumor (*black arrow*) and areas of necrosis (*yellow arrow*); 10× magnification. **b** 400× magnification of a section of the tumor showing necrotic and normal hepatocytes

**Fig. 2:**
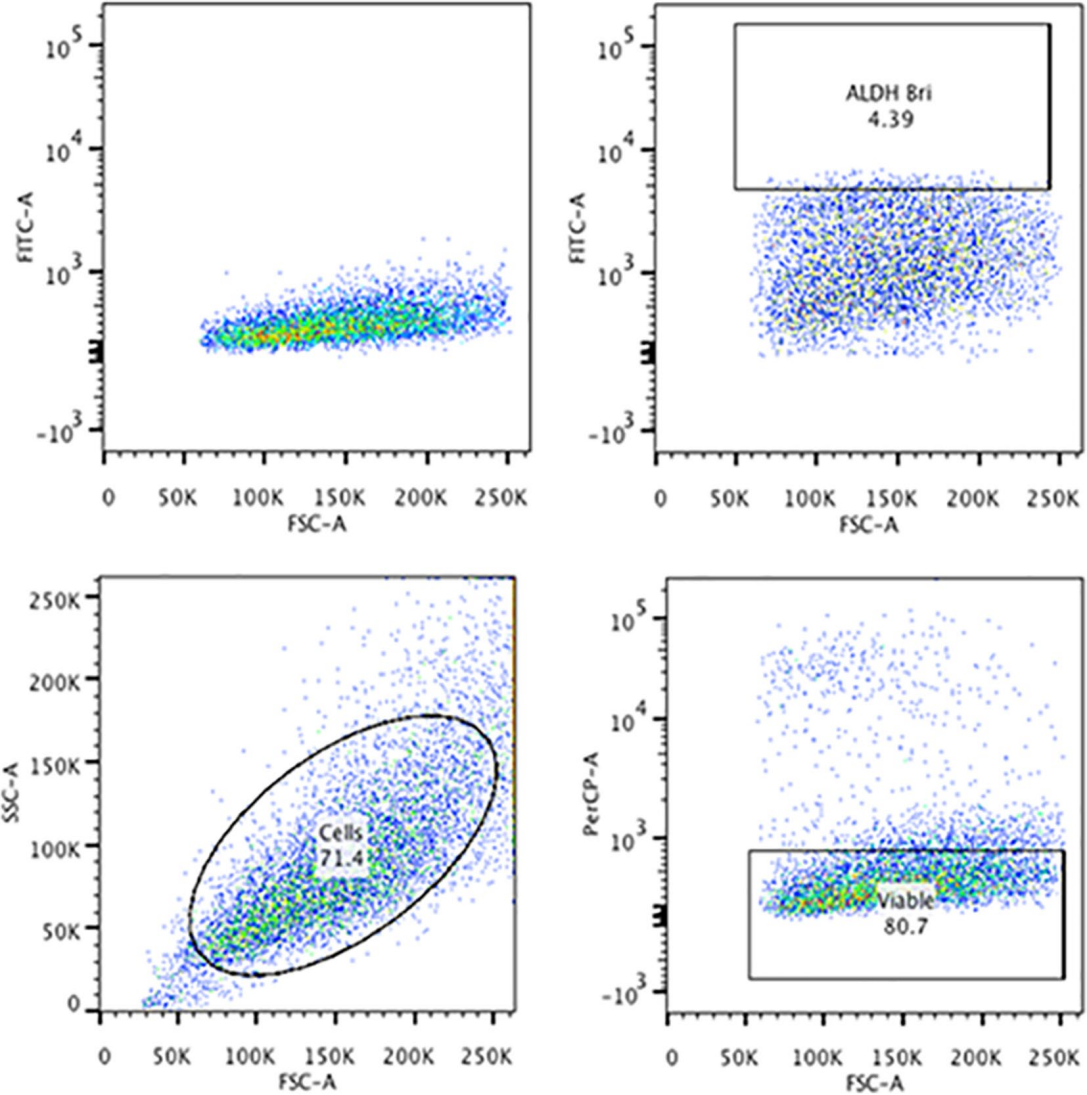
Aldefluor flow cytometry-based assay to identify cells with high ALDH1 activity in the rabbit VX2 liver cancer model. The* top panels* show the histograms of Aldefluor fluorescence with (*left*) and without (*right*) the addition of DEAB, an ALDH activity inhibitor. The gate demonstrates shifting of cells with high ALDH activity (*right*). The other two gates show the light scatter pattern and the viable cells based on PI exclusion. The gating strategy for DEAB and ALDH demostrated in Additional files 1 and 2

All the rabbits that were implanted with VX2 liver tumor cells developed solid tumors. Histological analysis of the masses using H&E staining revealed well differentiated atypical epitheliod tumors that were locally invasive and demonstrated choriodal distribution. These tumors are necrotic but have minimal inflammatory infiltration (Fig. 1a, b). We sorted tumor cells were sorted into different populations based on ALDH1 activity. The enzyme activity of the sorted ALDH-positive VX2 cell population was greater than 50 %, but for our study, we used the brightest 4.39 % of the total cancer cell population. Results are presented as percentages of ALDH bright cells or as ratios of mean fluorescence intensities (MFI) as compared with control cells incubated with DEAB (Fig. 2).

**Fig. 3:**
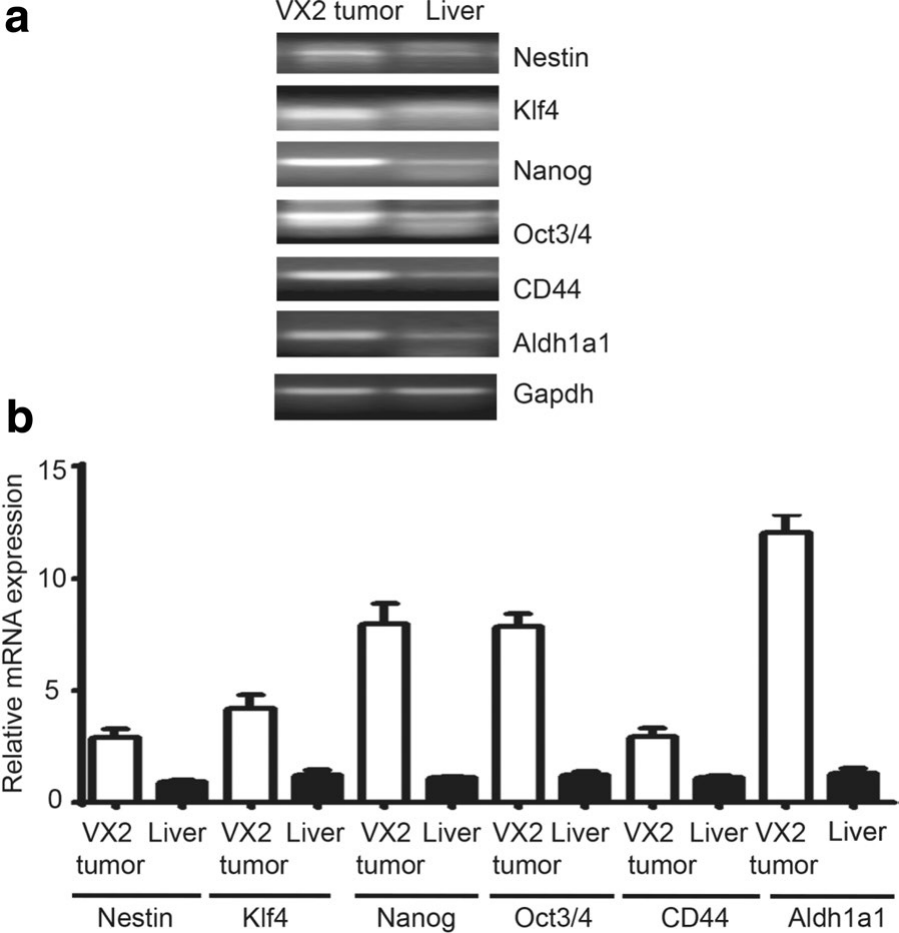
Analysis of mRNA expression. RNA were extracted from VX2 tumors and from normal rabbit liver cells as a control. **a** Semi-quantitative RT-PCR expression analysis revealed that VX2 tumors have higher levels of cancer stemness markers (nestin, klf4, nanog, oct3/4, Nanog,CD44 and Aldh1a1) in comparison to normal liver cells. **b** Real-time PCR using Ssofast Evagreen demonstrated similar trend of mRNA expression in VX2 tumors and in normal liver cells. Gapdh was used us control

**Fig. 4:**
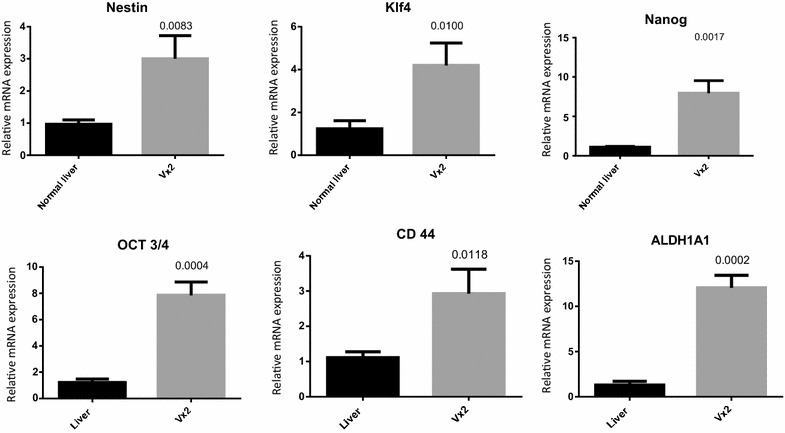
Group average expression of mRNA of stem cell markers (n = 3/group)

**Fig. 5:**
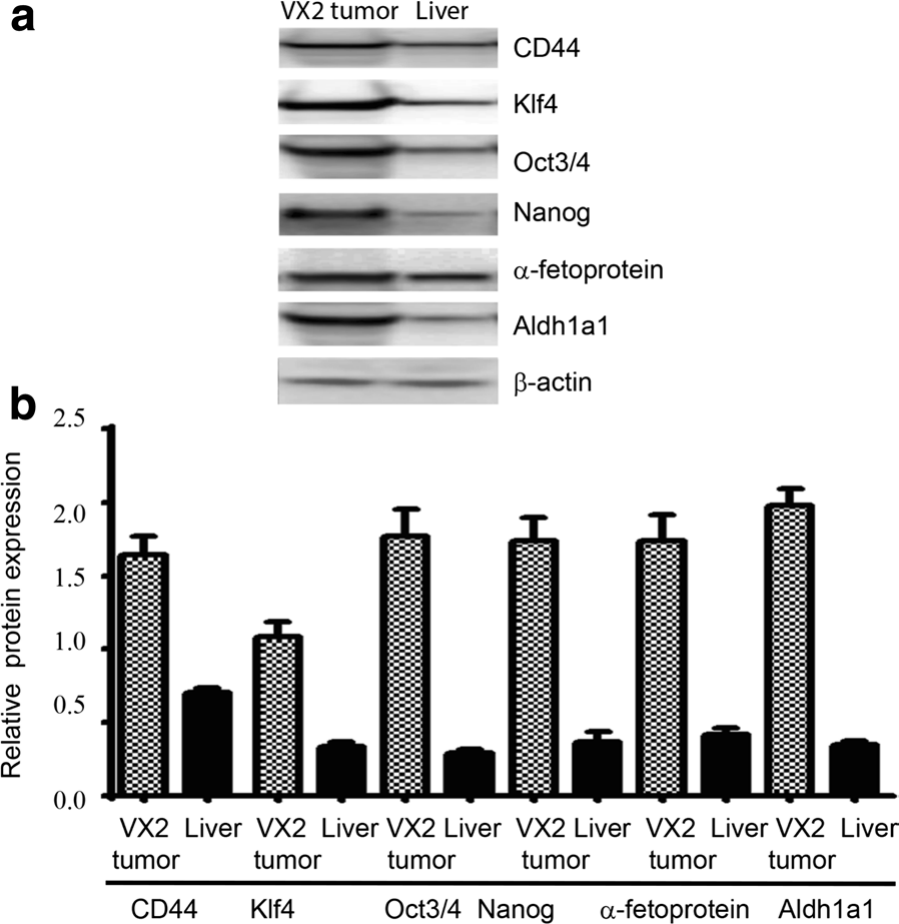
Analysis of protein expression by western blot. Proteins were extracted from VX2 tumors and from normal rabbit liver cells as a control. **a** β-actin expression was used as reference level. VX2 tumors expressed very high levels of stemness marker (CD44, klf4, oct3/4, nanog and Aldh1a1) in comparison to normal liver cells. Higher expression of cell differentiation marker α-fetoprotein in VX2 tumors in comparison to liver cells revealed that tumors from VX2 cells are well differentiated. **b** Quantification of protein expression in VX2 tumors and in normal liver cells

**Fig. 6:**
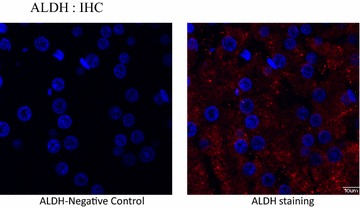
Immunoflourescent photomicrographs demonstrating cytoplasmic distribution of ALDH (*red*) in situ. The negative control imaged without the addition of the primary antibody

To characterize the stemness of derived VX2 tumors, we performed semi-quantitative as well as real time PCR on samples from the tumors and tissues from normal rabbit liver. We demonstrated that VX2 tumors have higher expression of cancer stem cell markers such as AlDH1A1 and CD44 in comparison to normal rabbit liver cells (Fig. 3). As these tumors are the first generation of tumors cells explanted from the rabbit, we speculated that the stem cell markers should be expressed significantly in these tumors. In accordance with the hypothesis, we verified that expression of stemness and pluripotent markers oct3/4, nanog and klf4 were overexpressed in VX2 tumors (Fig. 3). We also observed the overexpression of differentiation marker nestin in VX2 tumors in comparison to normal rabbit liver (Fig. 3). Additionally, real time PCR analysis of the same samples using syber-green demonstrated the significant change (p > 0.05) (Fig. 4) in the expression of the genes (Fig. 3). Next, we validated gene expression of the stemness markers by performing western blot (Fig. 5) and immunofluorescence (Fig. 6). We showed that cancer stem cell markers (AlDH1A1, CD44) and the differentiation marker α-fetoprotein were upregulated in VX2 tumor cells (Fig. 5). The same extent of upregulation was observed in stemness markers (klf4, oct3/4 and nanog) in VX2 tumors in comparison to normal rabbit liver (Fig. 5).

## Discussion

It has been established that CSCs play a critical role in the development of cancer. According to the CSC theory, not all cells in a tumor are equal; tumors are heterogeneous and many include the rare population with atavistic stem cell properties. These cells possess unique survival mechanisms and distinctive properties, such as the capacity to self-renew and differentiate, and also possess the capacity to proliferate following a prolonged period of quiescence. Our goal in these studies was to validate the VX2 orthotopic model by demonstrating the presence of CSCs in situ.

ALDH1 activity has been used as a functional stem cell marker to isolate CSCs in different types of cancers [36]. We found that ALDH1 was highly expressed in a 4.39 % population of cells in the rabbit VX2 liver cancer model, as determined using Aldefluor flow cytometry and validated with immunohistochemical and western blot analyses. IHC analysis demonstrated the localization of ALDH-positive cells in the cytoplasm. These findings suggest that ALDH1 is highly expressed in the cytoplasm of the rabbit VX2 tumor (Fig. 6). The frequency of expression of ALDH1 and the localization of the activity are consistent with many previous reports that demonstrate that the CSC population is small, usually accounting for only a fraction of all tumor types [37]. Collectively, the data produced in this study demonstrate that ALDH1 is a marker for VX2 CSCs. Characterization of the functionality of these markers will be performed in future studies in our laboratory.

Our findings show that an Aldefluor–FACS assay can successfully separate ALDH1-positive cells. Although more characterization of these cells is required, our work is a promising first step in development of a novel model for studying role of CSC in cancer therapy, resistance and recurrence. Because ALDH1-positive cells are viable and selectable on the basis of their fluorescence, they are readily available for stem cell studies. To the best of our knowledge, this is the first report for ALDH expression in the rabbit VX2 liver cancer model.

Expression of ALDH, especially the ALDH1A1 and ALDH3A1 isoforms, has been observed in normal cells as well as in CSCs. Expression seems to correlate with resistance to chemotherapy [38]. The high expression levels of ABC transporters and ALDH enzymes that have been found in CSCs suggest that these molecules can cooperate in the development of drug resistance in cancers. For example, ALDH1 overexpression has been positively associated with aggressive biological behavior of many cancer types, poor prognosis for patients, and drug resistance [7]. Therefore, the finding that ALDH1 overexpression in VX2 tumors promotes the idea that this model may be useful to study chemoresistance. However, further characterization will be necessary to validate the utility.

Our findings indicate that Aldefluor-based sorting of rabbit VX2 tumor cells could allow for CSC enrichment. They also support the validity of using the rabbit VX2 tumor model to study human hepatocellular carcinoma and provide concrete evidence that ALDH1 is expressed by and can be a functional marker in rabbit VX2 tumors. Our group has previously shown enhanced expression of ALDH1 in pig model of hepatocellular carcinoma using microarray expression and quantitative real time RT-PCR (Gehlot et al., unpublished). This novel finding that rabbit VX2 tumors have ALDH1 activity clarifies the utility of an important tool for understanding and identifying bipotential hepatic stem cells in a relevant large-animal model. Such understanding holds promise for development of new therapeutics for a wide range of liver pathologies, ranging from congenital metabolic diseases to end-stage liver cirrhosis to hepatocarcinogenesis. This newly characterized model should enhance the ability to rapidly translate future studies for clinical application.

## Conclusion

Our study results showed that we have identified and characterized the stemness of ALDH1A1 cells in rabbit VX2 liver cancer model. We demonstrated that VX2 tumors have higher expression of cancer stem cell markers such as AlDH1A1 and CD44 in comparison to normal rabbit liver cells. The purpose of this study is to summarize the role of stem like cell population in rabbit VX2 liver tumor to discuss current attempts of cancer stem cell targeting.

Most of the animal models currently available to study HCC are rodent models, which lack clinical relevance. This study represents the first report of ALDH activity in rabbit hepatic VX2 cancer model and demonstrates utility of the model as a tool for understanding and identification of bipotential hepatic stem cells in a relevant large-animal system. Based on these findings, we propose that the rabbit VX2 model may be useful for identifying new therapeutic treatments for a wide range of liver pathologies, including ranging from congenital metabolic diseases, end-stage liver cirrhosis, and hepatocarcinogenesis. The use of this model may enhance the translation of novel therapeutics for clinical application. Emerging therapeutic tools based on specific properties and functions of these subpopulations of cells inside the bulk of a tumor is crucial for improved clinical outcomes.

### Statistical analysis

Results were presented as the mean and standard error of the mean (SEM). Data analysis was with GraphPad Prism software version 6. Pairs of data were analyzed by unpaired t test assuming unequal variance. Statistical significance was defined as p < 0.05 unless stated otherwise.

## Additional files

10.1186/s12967-016-0785-0 The gating strategy used to define DEAB and ALDH positive cells.
10.1186/s12967-016-0785-0 The gating strategy used to define ALDH positive cells.
