# Supplementary figures and images for: Detection of ALDH1 activity in rabbit hepatic VX2 tumors and isolation of ALDH1 positive cancer stem cells
Source: J Transl Med. 2016 Feb 12;14:49. doi: 10.1186/s12967-016-0785-0 (PMC4752741; doi:10.1186/s12967-016-0785-0)

# FACSDiva Version 6.1.3

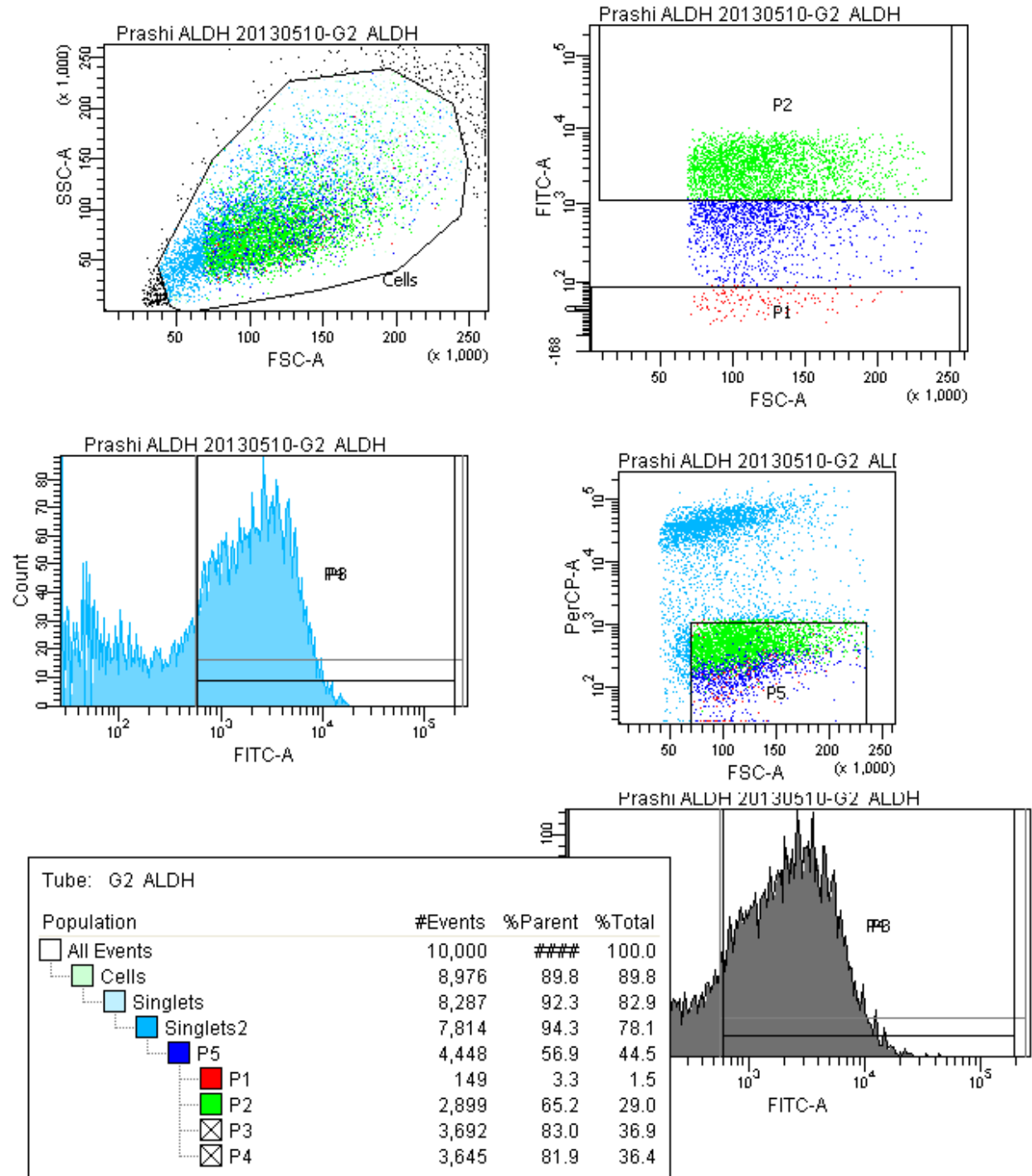

Supplement: Supplementary file 1 — 10.1186/s12967-016-0785-0 The gating strategy used to define DEAB and ALDH positive cells. [file 12967_2016_785_MOESM1_ESM.pdf]

# FACSDiva Version 6.1.3

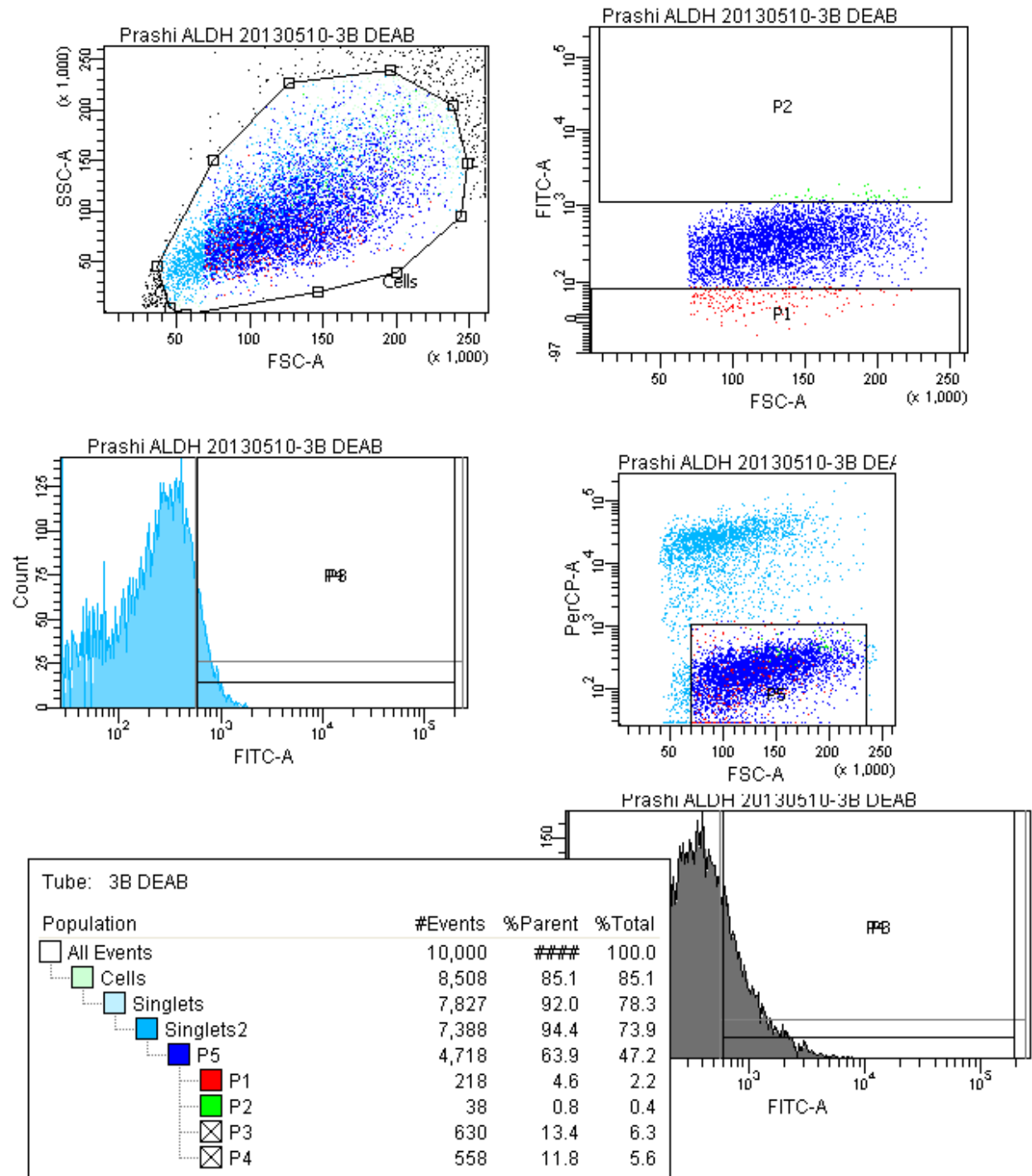

Supplement: Supplementary file 2 — 10.1186/s12967-016-0785-0 The gating strategy used to define ALDH positive cells. [file 12967_2016_785_MOESM2_ESM.pdf]
